# Summary of the best evidence for preventing the failure of autologous arteriovenous fistulas in maintenance hemodialysis patients

**DOI:** 10.3389/fcvm.2026.1872613

**Published:** 2026-07-08

**Authors:** Wei Qing, Zhaohua Zou, Jiquan Zhang, Xiu Hu, Ying Han

**Affiliations:** 1 Nephrology Department, Deyang People’s Hospital, Deyang, China; 2 School of Nursing, Chengdu University of Traditional Chinese Medicine, Chengdu, China

**Keywords:** autologous arteriovenous fistula, best evidence, evidence-Based nursing, failure, maintenance hemodialysis

## Abstract

**Background:**

Autologous arteriovenous fistula (AVF) is the preferred dialysis access for maintenance hemodialysis (MHD) patients and is regarded as the “lifeline” for these patients. A well-functioning AVF not only improves the adequacy of dialysis for the patients, but also enhances their quality of life.

**Objective:**

To retrieve, evaluate, and summarize the relevant evidence on preventing the failure of AVF in MHD patients, and to provide evidence-based basis for medical staff to formulate management measures.

**Methods:**

According to the “5S” pyramid model of evidence, search for relevant literature on AVF failure management on guideline websites, professional society websites, and Chinese-English databases. The types of literature include guidelines, expert consensuses, clinical decisions, evidence summaries, best practices, recommended practices, and systematic reviews. The search period is from the establishment of the database to January 20, 2026. Two researchers independently conducted literature quality evaluation, evidence extraction, and integration. The Joanna Briggs Institute (JBI) evidence pre-rating system of the Joanna Briggs Institute for Evidence-Based Health Care Center was used for evidence classification.

**Results:**

A total of 15 articles were included, including 6 guidelines, 4 expert consensuses, 4 clinical decisions, and 1 systematic review. 28 best evidence items were extracted from 6 aspects: including Personnel Management and Training, Perioperative Management, Intraoperative Management, Postoperative Management, Puncture Care, and Complications Management.

**Conclusion:**

This study summarizes the relevant evidence on preventing AVF failure in MHD patients and suggests that medical staff should formulate management plans for preventing AVF failure based on clinical scenarios.

**Systematic review registration:**

This study was registered at the Fudan University Centre for Evidence-based Nursing (Registration No. ES202610323).

## 1 Introduction

An epidemiological survey indicates that the number of patients with chronic kidney disease (CKD) worldwide reached 788 million in 2023, with a prevalence rate of 14.2%. The estimated number of patients with CKD in China is approximately 152 million (1). As global population aging intensifies, the number of patients progressing from CKD to end-stage renal disease (ESRD) is increasing year by year, requiring renal replacement therapy. Hemodialysis, peritoneal dialysis, and kidney transplantation are currently the main forms of renal replacement therapy. Over 90% of ESRD patients choose hemodialysis to remove water and toxins from the body and maintain life. According to data from the Chinese Hemodialysis Case Information Registration System, in 2024, there were approximately 1.027 million maintenance hemodialysis (MHD) patients in China, ranking first globally.

The prerequisite for hemodialysis treatment is the establishment of a reliable vascular access. The autologous arteriovenous fistula (AVF) is the lifeline for MHD patients and is currently recognized as the best vascular access, with advantages such as low infection risk, simple surgical operation, fewer complications, repeatable punctures, stable blood flow, and long lifespan. The functional status of AVF directly affects the dialysis efficiency and prognosis of patients (2). AVF failure occurs due to various reasons that cause internal fistula to become narrowed or occluded, resulting in a decrease or disappearance of blood flow, leading to insufficient dialysis or even inability to perform dialysis (3). According to the Expert Consensus on Vascular Access for Hemodialysis in China, one of the following three conditions can be used to determine AVF failure: ① Physical examination reveals that no tremor can be felt upon palpation, or the vascular murmur is weak or absent; ② The actual blood flow during dialysis is less than 200 mL/min; ③ Ultrasound examination reveals possible intimal hyperplasia and thickening or thrombosis in the blood vessels. Studies have shown that the 1-year, 3-year, and 5-year patency rates of AVF are 80.5%, 65.1%, and 50.5% (4), respectively. Its failure and related complications have become the primary cause of hospitalization for hemodialysis patients (5). The failure of AVF not only reduces the dialysis quality and quality of life of MHD patients, but sometimes even threatens their lives, increasing their pain and additional burden.

In clinical practice, medical staff lack clear decision-making basis for preventing AVF failure. The existing evidence is scattered and not systematic. This study employs the Evidence Summary methodological framework. Evidence Summary is an evidence synthesis method designed to address specific clinical questions by systematically searching for existing high-quality evidence (including systematic reviews, clinical guidelines, and expert consensus statements). After rigorous screening and evaluation, the evidence is synthesized and graded to generate concise, actionable clinical recommendations. This approach focuses on integrating existing comprehensive evidence to provide a rapid reference for clinical decision-making. Therefore, this study adopts evidence-based nursing methods, through systematic retrieval, strict evaluation, and comprehensive summary of the best evidence for preventing AVF failure management in MHD patients, aiming to provide a basis for clinical formulation of scientific management plans.

## 2 Materials and methods

### 2.1 Problem establishment

Based on the PIPOST model, an evidence-based question was constructed: population (P): MHD patients, aged ≥18 years; intervention (I): management strategies for preventing AVF failure; professional (P): medical staff and managers; outcome (O): relevant indicators of AVF failure, etc.; setting (S): hospital hemodialysis center and community; type of evidence (T): guidelines, expert consensus, systematic review, clinical decision, evidence summary, recommended practice, best practice.

### 2.2 Search strategy

Search Up To Date, BMJ Best Practice, the evidence-based health care international cooperation center library of the Australian Joanna Briggs Institute, Renal Association (RA), the National Institute for Health and Clinical Excellence (NICE), as guideline websites, the American National Kidney Foundation (NKF), the Global Initiative for Kidney Disease (KDIGO), etc. as society websites; PubMed, Web of Science, Embase, Cochrane Library, Chinese National Knowledge Infrastructure (CNKI), Wanfang Database, VIP Database, and China Biology Medicine disc(CBM) as Chinese and English databases. The search terms were “renal dialysis, renal dialyses, hemodialysis, hemodialyses, haemodialysis, extracorporeal dialysis, blood purification, blood dialysis, chronic kidney disease, chronic renal disease, chronic kidney failure, chronic renal failure, end-stage kidney disease, end-stage renal disease”, “fistula, arteriovenous fistula, vascular access, AVF”, “dysfunction, functional failure, functional impairment, risk factors, dangerous factors, influencing factors, affecting factors, predictive factors, management, prevention, complications”, “guidelines, consensus, systematic review, meta-analysis, clinical decision, evidence summary, recommended practice, best practice”; the search was conducted until January 20, 2026. Taking PubMed as an example, the search strategy is detailed in Table 1.

**Table 1:** Search strategy of pubmed (*n* = 15).

Search ID	Search formula
#1	“renal dialysis”[Mesh] OR “renal dialyses”[Title/Abstract] OR “hemodialysis”[Title/Abstract] OR “hemodialyses”[Title/Abstract] OR “haemodialysis”[Title/Abstract] OR “extracorporeal dialysis”[Title/Abstract] OR “blood dialysis”[Title/Abstract] OR “blood purification”[Title/Abstract] OR “chronic kidney disease”[Title/Abstract] OR “chronic renal disease”[Title/Abstract] OR “chronic kidney failure”[Title/Abstract] OR “chronic renal failure”[Title/Abstract] OR “end stage kidney disease”[Title/Abstract] OR “end stage renal disease”[Title/Abstract]
#2	“fistula”[Mesh] OR “arteriovenous fistula”[Title/Abstract] OR “vascular access”[Title/Abstract] OR “AVF”[Title/Abstract]
#3	“dysfunction”[Title/Abstract] OR “functional failure”[Title/Abstract] OR “functional impairment”[Title/Abstract] OR “risk factors”[Title/Abstract] OR “dangerous factors”[Title/Abstract] OR “influencing factors”[Title/Abstract] OR “affecting factors”[Title/Abstract] OR “predictive factors”[Title/Abstract] OR “management”[Title/Abstract] OR “prevention”[Title/Abstract] OR “complications”[Title/Abstract]
#4	“guideline”[Title/Abstract] OR “consensus”[Title/Abstract] OR “systematic review”[Title/Abstract] OR “meta-analysis”[Title/Abstract] OR “clinical decision”[Title/Abstract] OR “evidence summary”[Title/Abstract] OR “recommended practice”[Title/Abstract] OR “best practice”[Title/Abstract]
#5	#1 and #2 and #3 and #4

### 2.3 Inclusion and exclusion criteria

Inclusion criteria: The study population consists of adult patients undergoing MHD; the research content is the management strategies for preventing AVF failure; the research types cover guidelines, expert consensus, systematic reviews, clinical decision-making, evidence summary, recommended practices, best practices; the language is limited to Chinese or English. Exclusion criteria: Incomplete literature information or inability to obtain full text; failed quality assessment; brief versions of guidelines or guideline interpretation literature; duplicate guidelines; literature type is research plan or report.

### 2.4 Quality evaluation of the literature

Two researchers conducted the quality evaluation using a unified procedure. When opinions were inconsistent, the third researcher participated in the discussion to reach a final consensus. (1) Quality evaluation standard for guidelines: The 2017 version of the Clinical Guidelines Research and Evaluation System II (6) was used to evaluate the methodology of the guidelines, including 6 dimensions and 23 items. Each item was evaluated using a 7-level scoring method. (2) Quality evaluation standard for expert consensus: The Australian JBI Evidence-Based Health Care Center Expert Consensus Assessment Standard (2017) (7) was used to evaluate the methodological quality of the expert consensus, including 6 items. Each item was judged using “yes”, “no”, “unclear”, and “not applicable”. (3) Quality evaluation standard for systematic reviews: The JBI methodological quality evaluation tool for systematic reviews (7) was used for evaluation. The evaluation tool included 11 items, and each item was judged using “yes”, “no”, “unclear”, and “not applicable”. (4) Quality evaluation standard for clinical decision-making: Clinical decision-making is generally considered a high-level evidence type in the evidence grading system. Relevant evidence from the UpToDate Clinical Decision System, an authoritative evidence generation institution, is directly included as high-quality evidence. (5) Evaluation standard for evidence summary: The original quality rating of the evidence summary obtained from the JBI evidence database is directly used.

### 2.5 Evidence synthesis and grading

The researchers extracted the relevant information from the included literature. The main contents included the source of the literature, the publishing institution or author, the title, the research type, and the publication time. The researchers registered the included literature using a table, extracted the recommendations and research conclusions from each study, and formed items. Then, all the extracted contents were combined, merged the items with the same quality level (based on the higher quality level), and formed evidence summary. The evidence grade and recommendation strength were evaluated using the JBI 2014 Evidence Pre-Classification and Evidence Recommendation Level System (8). Grades 1 to 5 indicate from the highest to the lowest level. During the screening of similar evidence, when conflicts occur, high-quality and newly published evidence is preferred. The recommendation level of the evidence is determined according to the JBI FAME principle, taking into account F-feasibility, A-appropriateness, M-meaningfulness, and E-effectiveness. Evidence recommendations are classified as A-level recommendation (strong recommendation) and B-level recommendation (weak recommendation). Strong recommendation is made when the intervention measures clearly show that the benefits outweigh the drawbacks or the drawbacks outweigh the benefits, and it is supported by high-quality evidence. Otherwise, it is a weak recommendation.

## 3 Results

### 3.1 Literature search results and basic characteristics of the included studies

**Figure 1:**
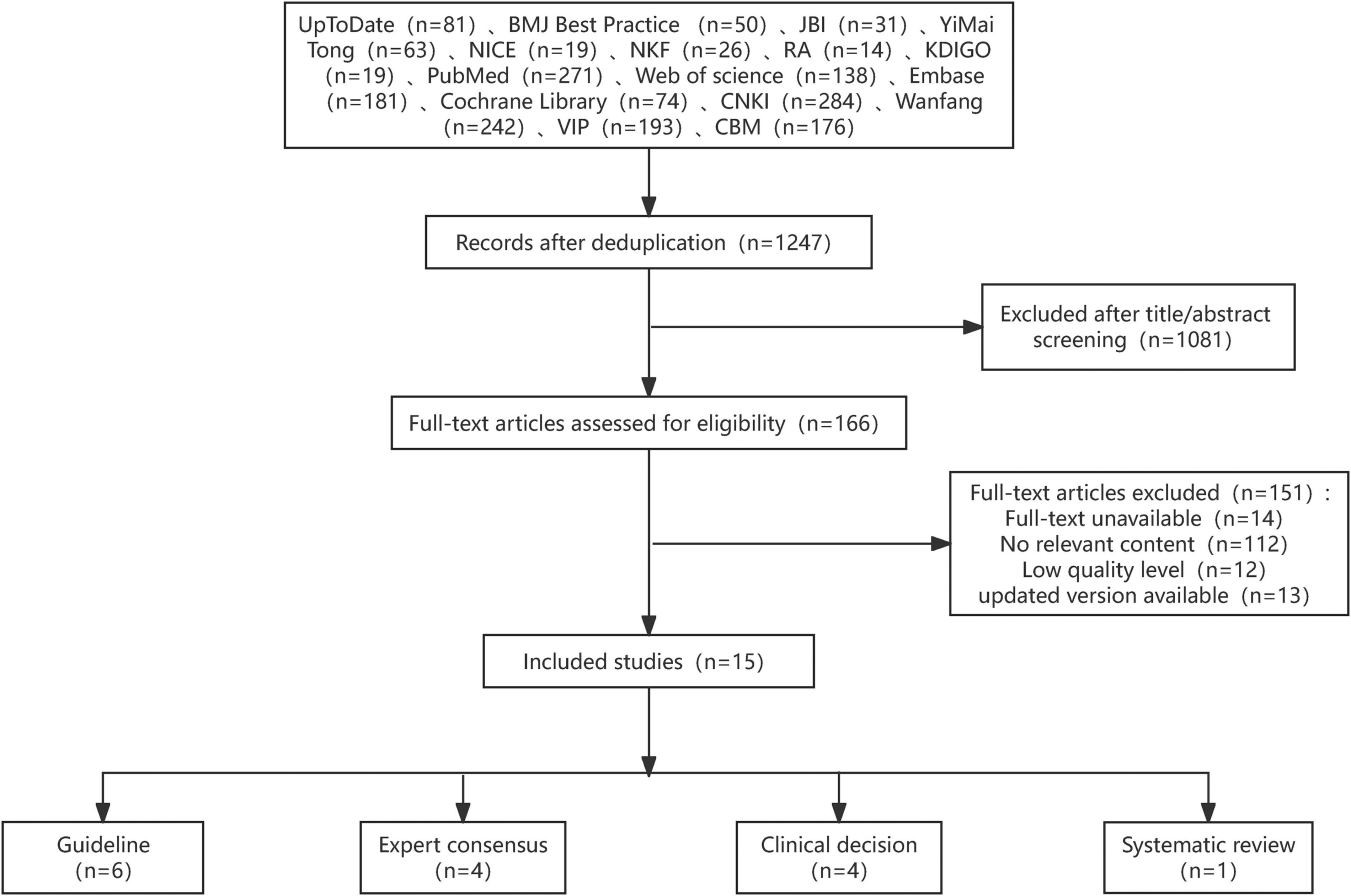
Flow chart of literature screening.

A total of 1,858 articles were retrieved, and finally 15 articles were included, including 6 guidelines, 4 expert consensuses, 4 clinical decisions and 1 systematic review. The literature screening process is shown in Figure 1, and the basic characteristics of the literature are presented in Table 2.

**Table 2:** Basic information of the included studies (*n* = 15).

Included studies	Publication year	Literature source	Literature theme	Literature type
Kukita et al. (9)	2015	Pubmed	Guidelines for Establishment and Repair of Hemodialysis Vascular Access	Guide
Gallieni et al. (10)	2020	Pubmed	Clinical Practice Guidelines for Perioperative and Postoperative Care of Arteriovenous Fistulas and Grafts in Adult Hemodialysis Patients	Guide
Lok et al. (11)	2020	American Kidney Foundation	KDOQI Clinical Practice Guidelines for Vascular Access	Guide
Schmidli et al. (12)	2018	Pubmed	Clinical Practice Guidelines for Vascular Access	Guide
Aitken et al. (13)	2025	Kidney Disease Association	Clinical Practice Guidelines for Hemodialysis Vascular Access	Guide
Chen et al. (14)	2024	Medical Pulse	Chinese Guidelines for Hemodialysis Vascular Access	Guide
Jin et al. (15)	2019	CNKI	Expert Consensus on Vascular Access for Hemodialysis in China	Expert consensus
Zhang et al. (16)	2023	CNKI	Expert Consensus on Perioperative Management of New Self-Transplanted Arteriovenous Fistulas	Expert consensus
Wan et al. (17)	2024	CNKI	Expert Consensus on Ultrasound Interventional Therapy for Vascular Access in Hemodialysis	Expert consensus
Xiao et al. (18)	2025	CNKI	Expert Consensus on Bedside Ultrasound Application in Hemodialysis Vascular Access Nursing	Expert consensus
Gerald (19)	2025	Up to date	Early Failure of Hemodialysis Arteriovenous Fistulas	Clinical decision
Gerald (20)	2024	Up to date	Failure of Mature Hemodialysis Arteriovenous Fistulas	Clinical decision
Gerald (21)	2024	Up to date	Risk Factors for Failure of Hemodialysis Arteriovenous Fistulas	Clinical decision
Karen (22)	2025	Up to date	Establishment of Hemodialysis Arteriovenous Fistulas and Their Complications	Clinical decision
Salih et al. (23)	2025	Pubmed	Predictive Factors for Early Failure of Arteriovenous Fistulas in Hemodialysis Patients	Systematic review

### 3.2 Literature quality assessment

#### 3.2.1 Quality assessment results of guidelines

This study retrieved 6 guidelines, among which 4 (10–13) were classified as level A, and 1 (14) as level B. All of them had high quality and were included. The results are shown in Table 3.

**Table 3:** Quality assessment results of guidelines (*n* = 6).

Included studies	Standardized percentage score by domain (%)						≥60%	≥30%	Recommendation (grade)
	Scope and purpose	People involved	Rigor of Development	Clarity of presentation	Applicability	Editorial independence			
Kukita et al. (9)	86	31	64	92	71	79	5	6	B
Gallieni et al. (10)	100	94	94	94	69	100	6	6	A
Lok et al. (11)	94	72	92	97	81	96	6	6	A
Schmidli et al. (12)	92	67	85	92	69	88	6	6	A
Aitken et al. (13)	92	92	68	89	69	100	6	6	A
Chen et al. (14)	92	64	57	67	56	83	4	6	B

#### 3.2.2 Quality assessment results of expert consensus

This study retrieved 4 expert consensuses (15–18), and all items were evaluated as “yes”. The quality was high, and all were included. The results are shown in Table 4.

**Table 4:** Quality assessment results of expert consensus (*n* = 4).

	Item					
Included studies	①	②	③	④	⑤	⑥
Jin et al. (15)	Yes	Yes	Yes	Yes	Yes	Yes
Zhang et al. (16)	Yes	Yes	Yes	Yes	Yes	Yes
Wan et al. (17)	Yes	Yes	Yes	Yes	Yes	Yes
Xiao et al. (18)	Yes	Yes	Yes	Yes	Yes	Yes

① Is the source of the opinion clearly identified? ② Does the source of opinion have standing in the field of expertise? ③ Are the interests of the relevant population the central focus of the opinion? ④ Is the opinion's basis in logic, experience, or underlying evidence clearly stated? ⑤ Is the argument or position presented logically? ⑥ Is there any incongruence with the available literature or evidence, and if so, is it logically defended?.

#### 3.2.3 Quality assessment results of clinical decision

This study retrieved 4 clinical decisions (19–22), all of which came from the UpToDate clinical decision system, and the quality was high. All were included.

#### 3.2.4 Quality assessment results of systematic reviews

This study retrieved 1 systematic review (23), and all items were evaluated as “yes”. The quality was high, and it was included. The results are shown in Table 5.

**Table 5:** Quality assessment results of systematic reviews (*n* = 1).

Included studies	①	②	③	④	⑤	⑥	⑦	⑧	⑨	⑩	⑪
Salih et al. (23)	Yes	Yes	Yes	Yes	Yes	Yes	Yes	Yes	Yes	Yes	Yes

① Is the clinical question clearly defined? ② Are the inclusion criteria for literature appropriate? ③ Is the search strategy appropriate? ④ Are the databases searched sufficient? ⑤ Are the criteria for assessing the quality of the literature appropriate? ⑥ Is at least a two-person independent (back-to-back) evaluation conducted? ⑦ Are measures taken to reduce errors during data extraction? ⑧ Are the methods used to combine studies appropriate? ⑨ Is the possibility of publication bias assessed? ⑩ Are the recommendations based on the results of the systematic review? ⑪ Is the direction for further research appropriate?.

## 4 Summary of evidence

By summarizing the evidence for preventing the failure of AVF, 28 pieces of evidence were summarized from 6 aspects: Personnel Management and Training, Perioperative Management, Intraoperative Management, Postoperative Management, Puncture Care, and Complications Management. As shown in Table 6.

**Table 6:** Summary of evidence on preventing the failure of arteriovenous fistula.

Category	Evidence summary	Evidence level	Recommendation level
Personnel Management and Training	1. Establish a multidisciplinary team for hemodialysis vascular access, consisting of: nephrologists, dialysis nurses, vascular access doctors, radiologists, and access coordinators. (14)	2	A
	2. Establish a systematic vascular access training system to ensure that vascular access doctors, nurses, and coordinators all receive standardized training, so as to effectively monitor and promptly detect blood flow dysfunction of the AVF access. (11)	5	B
	3. Carry out high-quality training on venipuncture, and regularly update the training content to maintain the operational ability for arteriovenous fistula puncture. (11)	5	B
Perioperative management	4. The risk factors for the failure of AVF establishment should be fully evaluated, including gender, advanced age, obesity, diabetes, arteriosclerosis, malignant tumors, neurological and mental disorders, skin diseases, and previous history of arteriovenous operations. (16)	5	B
	5. The vascular access specialist should comprehensively utilize methods such as visual examination, palpation, and ultrasound examination to conduct a systematic assessment of the patient's forearm arteries and veins; for those suspected of having vascular access dysfunction, ultrasound examination should be the first choice. Detailed records of the vascular course should be made, and a vascular access construction plan should be formulated. (9, 12)	3	A
	6. For CKD patients (G3-G5) who are undergoing dialysis or are expected to require dialysis in the future, efforts should be made to protect their central veins and peripheral arteriovenous shunts from damage, including avoiding the use of peripheral inserted catheters and unnecessary venous punctures. (11)	5	B
	7. Instruct the patients to start performing functional exercises for the upper limb on the surgical side 4 weeks before the AVF operation, in order to increase the inner diameter of the blood vessels and blood flow, thereby enhancing the success rate of the surgery. (16)	1	A
Intraoperative management	8. It is recommended to adopt the arterial side—venous end anastomosis method. This surgical approach can effectively prevent distal venous hypertension while not affecting the vascular patency rate. (9)	5	B
	9. When performing AVF establishment surgery for ESRD patients, regional block anesthesia is preferred over local anesthesia, which helps to increase the postoperative patency rate of the vascular access. (14, 22)	1	A
	10. If the patient has a central venous catheter in place or a pacemaker implanted, a vascular access should be established on the opposite upper limb to reduce the risk of central venous stenosis and decreased patency of the access. (12)	3	A
	11. The veins should be left with an appropriate length, the unnecessary branches should be ligated, and the blood vessels should be rinsed with heparinized normal saline and expanded, thereby improving the success rate and long-term patency rate of AVF surgery. (16)	5	B
Postoperative management	12. After AVF surgery, patients should be taught to monitor the patency of the blood vessel themselves. The patients should be instructed to check the function of the newly established internal fistula by feeling for tremors. If there is no tremor, they should determine it by listening for sounds. (16)	3	A
	13. After the establishment of the AVF, instruct the patient to carry out the planned functional exercises for the surgical arm and adopt far-infrared therapy to promote the maturation of the internal fistula and improve the long-term patency of the arteriovenous fistula. (10)	1	A
	14. Four to six weeks after the surgery, experienced medical personnel should assess the maturity of the AVF. If it is still not mature after six weeks, a further color Doppler ultrasound examination should be arranged immediately for evaluation. (14, 19)	3	A
	15. Strengthen the management of blood pressure during the perioperative period and the maturation stage of the internal fistula to prevent hypotensive events, and regularly assess the patient's medication regimen and dry body weight setting. (13)	3	A
Puncture care	16. The rope ladder puncture method is regarded as the preferred puncture technique for AVF. (11)	1	A
	17. The initial AVF catheterization for the patients is performed by nurses with proficient puncture skills and high success rates, in order to avoid the primary mechanical damage to the AVF catheterization site. (11)	5	B
	18. When necessary, bedside ultrasound trained by standardized methods can be used by nurses to assist in the puncture operation of complex AVF, in order to increase the success rate and safety of the puncture, and prevent iatrogenic vascular damage. (18)	1	A
	19. After the dialysis procedure, the puncture needle should be completely withdrawn before immediately applying pressure to stop the bleeding. The pressure should be moderate, ensuring that it can effectively stop the bleeding without blocking the blood flow and still allowing for the detection of vascular tremors. In cases where necessary, bedside ultrasound-assisted AVF compression hemostasis technique can be employed. (18)	3	A
Complications Management	20. Identify the risk factors for AVF failure, including: hemodynamic factors, patient-related demographic characteristics or clinical factors, as well as technical operation-related factors. (21, 23)	3	A
	21. Before each dialysis session, experienced medical staff should conduct a physical examination of the AVF to detect potential complications at an early stage. For patients with clinically evident AVF stenosis, vascular angioplasty is preferred to reduce the risks of thrombosis and internal fistula failure through further imaging examinations. (15, 20)	4	B
	22. It is recommended to use ultrasound-guided brachial plexus nerve block anesthesia as the preferred anesthesia method for PTA surgery. (17)	4	B
	23. The following situations may warrant the use of stents, and covered stents are recommended: residual stenosis exceeding 50% after balloon dilation, recurrence of stenosis within a short period of time, severe dissection or pseudoaneurysm during the operation, etc. (17)	1	A
	24. The treatment method for saving the thrombosed vascular pathway should be determined based on the patient's condition, the status of the vascular access, and the local medical technical conditions, and a combination of interventional therapy via the blood vessel or surgical operation should be considered. (10)	1	A
	25. If a thrombus occurs within 7 days after the establishment of an arteriovenous fistula, thrombolytic therapy is not recommended. (14)	4	B
	26. In the following situations, surgical treatment should be chosen for thrombosis: when endovascular treatment fails, when the lesion is too severe to be treated endovascularly, or when surgical treatment is expected to have a better outcome. (11)	5	B
	27. Physical examination is the primary method for assessing infections, while imaging examinations can serve as an auxiliary diagnostic basis for AVF infections. (14)	5	B
	28. For infectious vascular access, empirical broad-spectrum antibiotic treatment should be initiated promptly. Close monitoring of the results of pathogen culture is necessary, and the antibiotic regimen should be adjusted in a timely manner based on the susceptibility test. The patient should also be referred to a surgeon who is familiar with the management of vascular access complications for further treatment. (11)	5	B

## 5 Discussion

Compared with arteriovenous grafts and central venous catheters, AVF has fewer complications, higher patency, and a longer survival period. It is the preferred vascular access recommended by the guidelines for the quality of life of patients with kidney diseases. However, the maturity and patency rates of AVF are not satisfactory. Therefore, by comprehensively summarizing the best evidence for managing AVF failure in MHD patients, it can provide guidance for clinical practice. Integrated management of vascular access involves the integration and application of multiple technical methods. Besides physical examination and routine care, it also requires the use of ultrasound and radiological intervention techniques for comprehensive assessment and treatment. This determines that the vascular access team must be composed of professionals from multiple disciplines. Team members should be involved in all aspects of establishing, evaluating and monitoring the vascular access, as well as handling complications. Raza et al. (24) found that the standardized maintenance of the vascular access team can achieve early identification of complications and have a positive impact on the long-term prognosis of the access. The access coordinator, as the core of the vascular access team, mainly undertakes the communication and coordination duties between patients and various professionals. This position should be held by senior dialysis nurses or physicians with more than 2 years of experience in vascular access. Compared to having clinical physicians undertake this task concurrently, appointing a dedicated access coordinator often has more advantages. It not only possesses excellent communication and coordination skills but also can more professionally conduct patient education and daily guidance and management of the vascular access. The KDOQI guidelines (11) clearly propose to implement a systematic training program and continuous quality monitoring for vascular access staff, and update the training content regularly to consolidate the staff's theoretical foundation and improve the puncture operation level, achieving the goal of controlling infections and reducing complications. Simulation teaching has the characteristics of standardization and controllability. Trainees can repeatedly practice the standard operation procedures under zero-risk conditions, and teachers can promptly identify and correct the trainees’ mistakes. A meta-analysis that included 7 RCT studies confirmed that compared to the traditional teaching mode, simulation teaching can significantly improve the success rate of vascular access operations (25). It should be noted that currently, the establishment of a systematic vascular access training system and the provision of standardized training are mostly based on expert consensus. Their applicability may vary depending on different medical resource conditions.

AVF, as the preferred vascular access type for MHD patients, the quality of its preoperative management directly determines the maturity rate, lifespan and complication rate of the internal fistula, and is the primary step to ensure the successful establishment of the internal fistula. A RCT indicated that the success rate of AVF in the group that received preoperative ultrasound examination was approximately 19% higher than that in the group without examination (26). Additionally, a meta-analysis suggested that routine preoperative Doppler ultrasound screening has greater clinical value than selective screening, significantly reducing the early loss of AVF (27). Another study indicated that preoperative vascular tracing technology helps improve the success rate of surgery and the long-term patency rate of AVF (28); for patients with high-risk factors for AVF failure, it is recommended to complete preoperative vascular tracing under ultrasound guidance (29). At the level of intraoperative management, A multicenter randomized controlled trial confirmed that compared with local anesthesia, brachial plexus block anesthesia significantly improved the patency rate and functional patency rate of AVF, and had a better cost-effectiveness ratio (30); at the same time, a meta-analysis also showed that regional block anesthesia helped to improve the initial patency rate of AVF and local blood flow (31). Although the above evidence comes from randomized controlled trials and systematic reviews, and is of a relatively high level in the existing literature, its limitations still need to be noted: The follow-up period of the above RCTs and the burden of comorbidities of the included population may not fully represent the extensive patient population in the real world, and the heterogeneity in the methods of block anesthesia, drug dosage, and outcome definition in the meta-analysis may overestimate the net benefit of regional block. General anesthesia is rarely used in clinical practice due to its complex operation and high risks; for patients who cannot cooperate, intravenous anesthesia can be considered after comprehensive assessment by the anesthesiologist. However, there is a lack of direct comparative data from large-scale randomized controlled trials regarding the applicable scenarios and risk avoidance strategies of general anesthesia and intravenous anesthesia. The currently recommended clinical anesthesia protocol is: When ultrasound guidance is available, priority should be given to using brachial plexus nerve block; when there is no such condition, local infiltration anesthesia should be selected. General anesthesia is not a routine choice. This recommendation is based on the integration of the best available evidence. Moreover, most of the above RCTs were conducted in specific medical centers, and their technical conditions (such as the configuration of ultrasound equipment, the operational experience of anesthesiologists) and patient characteristics may differ from those in grassroots or resource-limited areas, which limits the widespread promotion of the recommended protocol to different levels of medical institutions.

After AVF surgery, intensified monitoring and standardized management are necessary. Within 4 h after the operation, the vascular patency should be monitored once every hour. From then on, it should be monitored at least once a day (14). At the same time, patients should be instructed to master the self-monitoring method. If any abnormalities are found, they must be reported to the medical staff immediately. It is necessary to clearly inform patients that they are prohibited from lifting heavy objects and performing medical operations on the arm with the internal fistula. After the operation, appropriately elevating the surgical side arm can help reduce limb edema. The maturity of the AVF should be evaluated by the vascular access team 4–6 weeks after the operation. The incidence of AVF maldevelopment is relatively high, approximately 25%–70% (32). To promote the maturation of the internal fistula, the KDOQI guidelines (11) recommend formulating a standardized arm movement plan and urging patients to complete it: guide finger flexion exercises on the first day after the operation; perform fist clenching exercises on the third day after the operation; start pressurized fist clenching/holding ball exercises 2 weeks after the operation or when the wound is completely healed. A meta-analysis has confirmed that the arm movement training program can effectively improve the maturity of AVF and ensure the normal use of the access (33), this is because performing light fist-clenching movements on the operated limb helps to alleviate vascular spasms. In addition, a meta-analysis has confirmed that far-infrared radiation therapy can promote the maturation of the internal fistula, shorten the hospital stay, and reduce the rate of AVF occlusion (34)，Far-infrared rays are an invisible type of electromagnetic wave, with a longer wavelength than visible light, ranging from 5.6 to 1,000.0 micrometers. Studies have shown that far-infrared ray irradiation therapy can inhibit inflammation, suppress intimal hyperplasia, reduce oxidative stress, improve endothelial function, increase blood flow in the internal fistula, and promote the maturation of the internal fistula. For those with maldevelopment, ultrasound-guided balloon-assisted maturation technology can be used. A systematic review has confirmed its significant clinical effect (35)^.^ The above evidence is reliable. When applying it in clinical practice, it can be combined with specific circumstances and patient needs to formulate a standardized nursing implementation process.

The lifespan of an AVF depends on multiple factors such as the patient's vascular condition, surgical skills, and puncture techniques. Standardized puncture procedures are crucial for prolonging the usage period of the internal fistula (36). The vascular access team needs to comprehensively assess the vascular direction, elasticity, and effective length of the patient's AVF before puncture and develop an individualized puncture plan. Nurses, as the direct users of the internal fistula, need to assess the function of the access before each puncture and play a key role in vascular access maintenance. The Dialysis Outcomes and Practice Patterns Study (DOPPS) found that for every 20% increase in the number of experienced nurses (those with more than 3 years of experience in hemodialysis), the power loss of the AVF can be reduced by 11%, effectively avoiding primary mechanical damage to the access (12). Rope ladder puncture, the puncture point is more than 3 cm away from the internal fistula anastomosis. At the arterial end and venous end, at least 3 non-fixed puncture points are set, with a distance of more than 0.5 cm between each point. This puncture technique can prevent localized vascular stenosis, tumor-like dilation and thrombosis, and prolong the service life of the internal fistula. In terms of puncture technique selection, the rope ladder puncture technique has always been regarded as the standard method and should be the preferred puncture technique. The eyelet puncture technique has advantages in difficult AVF punctures and can increase the puncture success rate, but some studies have reported that its infection risk is significantly higher than the rope ladder puncture, so it should only be used as a supplementary option, applicable to special situations such as short puncture segments of AVF, large or progressive expanding aneurysms that need to be prevented from further expansion, etc. For complex AVFs, ultrasound real-time guidance puncture is recommended (37). A meta-analysis has shown that this technique can increase the success rate of the first puncture and reduce the incidence of post-puncture complications (38). In addition, bedside ultrasound can also be applied to precise compression hemostasis of AVF. The ultrasound-guided compression technique has the advantages of real-time imaging, high sensitivity, and precise positioning, and can clearly display the overflow of color blood flow signals at the puncture point, thereby providing reliable imaging support for compression hemostasis (18). Although ultrasound-guided puncture can reduce the failure rate of puncture and minimize vascular damage, its promotion is constrained by the operator's dependence and resource limitations. This technique requires operators to possess the combined skills of ultrasound image interpretation and real-time guidance. The training period is long and the standardization is insufficient, making it difficult for grassroots medical institutions to replicate. Moreover, portable ultrasound equipment has high purchase costs and strict maintenance requirements, and its accessibility is limited in resource-constrained areas. Therefore, this study sets its recommendation strength at level B, suggesting that it should be implemented preferentially in centers with the necessary conditions. Grassroots medical institutions can make up for the technical gap through medical alliance cooperation or referral mechanisms.

The risk factors for AVF failure mainly include four aspects: hemodynamic factors (venous diameter, diameter of the supplying artery and blood flow), patient-related factors (age, gender, ethnic background and other demographic characteristics), clinical comorbidities (cardiac diseases, peripheral artery diseases, pulmonary hypertension, diabetes, obesity, etc.) and technical operation factors (training and experience of the surgeon, establishment, care and usage norms of AVF. Among them, hemodynamic factors are the most critical. Demographic characteristics and clinical risk factors usually only play a role when they affect hemodynamics (22). Therefore, in clinical practice, it is necessary to pay close attention to these risk factors and take active preventive measures. In addition to regular monitoring, before each puncture, experienced medical staff should conduct a physical examination through “visual, tactile and auditory” methods. If any abnormalities are found, further imaging assessment should be carried out; if there are clinical manifestations, intervention should be carried out promptly. In addition, the arm-raising test and the pulsation enhancement test are also important methods and means for clinical nurses to assess the function of patients' AVF. They have the characteristics of rapid and efficient screening.

Stenosis represents the most prevalent complication of AVF, with the potential to impair maturation, compromise access function, reduce dialysis adequacy, precipitate thrombotic occlusion, and ultimately result in access loss. Contemporary therapeutic modalities encompass both endovascular interventions and surgical procedures. In recent years, endovascular approaches—specifically percutaneous transluminal angioplasty (PTA) and stent implantation—have supplanted surgical revision as the preferred first-line treatment. PTA constitutes a safe and effective technique capable of achieving immediate vessel recanalization, restoring AVF function, and preserving vascular resources. The KDOQI guidelines (11) endorse high-pressure balloon PTA as the treatment of choice for AVF stenosis, with dilation procedures repeatable until satisfactory luminal patency is achieved. Key technical considerations include systemic heparinization prior to intervention and strict limitation of single-balloon inflation duration to 30–60 s to mitigate the risk of acute thrombosis, while minimizing blood flow interruption. Thrombosis constitutes another major cause of AVF dysfunction, accounting for the permanent abandonment of 65%–85% of accesses (14). Although thrombolytic techniques can achieve thrombus dissolution, they are associated with prolonged treatment duration, elevated bleeding risk, and significant pulmonary embolism incidence; moreover, long-term patency remains suboptimal due to the failure to address underlying concurrent stenosis. Indeed, endothelial hyperplasia–induced vascular narrowing represents the fundamental pathophysiological mechanism of thrombosis, with over 85% of thrombotic events accompanied by stenosis (39). Thrombolytic therapy for AVF thrombosis in patients receiving MHD remains controversial. Current evidence suggests that pharmacological thrombolysis is effective primarily for fresh thrombi; however, this conclusion derives predominantly from retrospective studies and clinical experience, lacking validation by prospective controlled trials, thereby limiting its certainty. Systemic thrombolysis is generally discouraged due to heightened bleeding risk, though this recommendation similarly lacks direct support from large-scale randomized controlled trials. In contemporary practice, beyond local urokinase thrombolysis, mechanical aspiration thrombectomy, balloon-assisted thrombectomy, and surgical thrombectomy have gained increasing acceptance, with concurrent treatment of vascular stenosis upon thrombus removal emphasized to prevent recurrence. Nevertheless, these recommendations rely principally on observational data and consensus statements, constraining causal inference strength. Furthermore, the primary studies were predominantly conducted in centers with mature interventional infrastructure, potentially diminishing generalizability to resource-constrained settings. Future research should prioritize well-designed, adequately powered randomized controlled trials to compare treatment modalities, elucidate the incremental benefit of concurrent angioplasty, and thereby elevate both the evidence grade and universal applicability of clinical recommendations.

## 6 Conclusion

This study systematically summarized the best evidence for preventing AVF failure in MHD patients, including Personnel Management and Training, Perioperative Management, Intraoperative Management, Postoperative Management, Puncture Care, and Complications Management. It provides reliable evidence-based basis for clinical prevention of AVF failure. It is recommended that during the process of evidence transformation, the technical conditions of the medical institution, the patient's wishes, and the status of the vascular access should be fully considered to formulate individualized prevention and management plans for AVF failure.

## 7 Limitations

This study is subject to several limitations that warrant careful consideration. First, a subset of the recommendations presented herein is derived from guideline-derived and consensus-based evidence, which inherently carries a lower certainty of effect estimates and limited recommendation strength; consequently, these recommendations should be interpreted with appropriate caution in clinical practice. Second, the present review partially incorporated tertiary clinical resources from UpToDate, which were utilized primarily to inform clinical decision-making. It should be acknowledged that UpToDate constitutes an integrated, narrative review–type resource rather than primary evidence or official guidelines; as such, it is susceptible to the database update cycle and the subjective synthesis of contributing experts, resulting in a comparatively low level of evidence. Future updates to this review should prioritize the substitution of these lower-tier sources with high-quality randomized controlled trials and newly published authoritative guidelines as they become available. Third, the body of clinical literature addressing the early prevention of AVF failure in patients receiving MHD remains limited. Future research should prioritize well-designed, adequately powered randomized controlled trials to rigorously evaluate the preventive efficacy of specific interventions against AVF failure in this patient population, thereby strengthening the evidence base underpinning clinical recommendations.

## References

[1] GBD 2023 Chronic Kidney Disease Collaborators. Global, regional, and national burden of chronic kidney disease in adults, 1990–2023, and its attributable risk factors: a systematic analysis for the global burden of disease study 2023. Lancet. (2025) 406(10518):2461–82. 10.1016/S0140-6736(25)01853-7 41213283

[2] Ravani P Quinn RR Oliver MJ Karsanji DJ James MT MacRae JM. Preemptive correction of arteriovenous access stenosis: a systematic review and meta-analysis of randomized controlled trials. Am J Kidney Dis. (2016) 67(3):446–60. 10.1053/j.ajkd.2015.11.013. Erratum in: Am J Kidney Dis. (2017) 70(5):735. doi: 10.1053/j.ajkd.2017.07.006. 26776537

[3] Rajabi-Jaghargh E Banerjee RK. Combined functional and anatomical diagnostic endpoints for assessing arteriovenous fistula dysfunction. World J Nephrol. (2015) 4(1):6–18. 10.5527/wjn.v4.i1.6 25664243 PMC4317629

[4] Shrestha PC Asher J Shrestha SM Jenner S Wilson C Taylor C. Survival of arteriovenous fistula for dialysis at different centers in the north of England. J Vasc Access. (2007) 8(4):231–4. 10.1177/112972980700800403 18161667

[5] Pisoni RL Zepel L Port FK Robinson BM. Trends in US vascular access use, patient preferences, and related practices: an update from the US DOPPS practice monitor with international comparisons. Am J Kidney Dis. (2015) 65(6):905–15. 10.1053/j.ajkd.2014.12.014 25662834

[6] AGREE Next Steps Consortium. The AGREE II Instrument [EB/OL]. (2017). Available online at: https://www.agreetrust.org/wp-content/uploads/2017/12/AGREE-II-Users-Manual-and-23-item-Instrument-2009-Update-2017.pdf (Accessed December 31, 2021).

[7] Hu Y Hao YF. Evidence-based Nursing. 2nd ed. Beijing: People’s Medical Publishing House (2018). p. 72–84.

[8] Wang CQ Hu Y. JBI evidence pre-grading and evidence recommendation level system (2014 edition). J Nurs Educ. (2015) 30(11):964–7. 10.16821/j.cnki.hsjx.2015.11.002

[9] Kukita K Ohira S Amano I Naito H Azuma N Ikeda K. 2011 update Japanese society for dialysis therapy guidelines of vascular access construction and repair for chronic hemodialysis. Ther Apher Dial. (2015) 19(Suppl 1):1–39. 10.1111/1744-9987.12296 25817931

[10] Gallieni M Hollenbeck M Inston N Kumwenda M Powell S Tordoir J. Clinical practice guideline on peri- and postoperative care of arteriovenous fistulas and grafts for haemodialysis in adults. Nephrol Dial Transplant. (2020) 35(12):2203. 10.1093/ndt/gfaa106 32365363

[11] Lok CE Huber TS Lee T Shenoy S Yevzlin AS Abreo K. KDOQI clinical practice guideline for vascular access: 2019 update. Am J Kidney Dis. (2020) 75(4 Suppl 2):S1–164. 10.1053/j.ajkd.2019.12.001 32778223

[12] Schmidli J Widmer MK Basile C de Donato G Gallieni M Gibbons CP. Editor’s choice—vascular access: 2018 clinical practice guidelines of the European society for vascular surgery (ESVS). Eur J Vasc Endovasc Surg. (2018) 55(6):757–818. 10.1016/j.ejvs.2018.02.001 29730128

[13] Aitken E Anijeet H Ashby D Barrow W Calder F Dowds B. UK Kidney association clinical practice guideline on vascular access for haemodialysis. BMC Nephrol. (2025) 26(1):461. 10.1186/s12882-025-04374-y 40813633 PMC12351868

[14] Expert Group of Nephrology Branch, Chinese Medical Association. Chinese guideline for dialysis access (2024 edition). Chin J Nephrol. (2024) 40(12):990–1070. 10.3760/cma.j.cn441217-20230926-00935

[15] Vascular Access Working Group, Blood Purification Center Branch, Chinese Hospital Association. Expert consensus on vascular access for hemodialysis in China (2nd edition). Chin J Blood Purif. (2019) 18(6):365–81. 10.3969/j.issn.1671-4091.2019.06.001

[16] Zhang DL, Expert Consensus Working Group of Nephrology and Blood Purification Branch, Beijing Perioperative Medicine Research Association. Expert consensus on perioperative management of newly created autogenous arteriovenous fistula. Chin J Blood Purif. (2023) 22(12):881–90. 10.3969/j.issn.1671-4091.2023.12.001

[17] Expert Group on Consensus for Ultrasound-guided Interventional Therapy of Vascular Access for Hemodialysis in China. Expert consensus on ultrasound-guided interventional therapy of vascular access for hemodialysis in China (2024 edition). Chin J Nephrol. (2024) 40(11):918–30. 10.3760/cma.j.cn441217-20240513-00510

[18] Blood Purification Professional Committee of Beijing Nursing Association, Beijing Nursing Quality Control and Improvement Center, Beijing Haidian Hospital (Haidian Campus of Peking University Third Hospital). Expert consensus on bedside ultrasound application in vascular access nursing for hemodialysis (2025 edition). Chin J Mod Nurs. (2025) 31(34):4621–35. 10.3760/cma.j.cn115682-20250701-03465

[19] Gerald AB. Primary failure of the hemodialysis arteriovenous fistula. Available online at: https://www.uptodate.cn/contents/zh-Hans/primary-failure-of-the-hemodialysis-arteriovenous-fistula? (Accessed February 25, 2026).

[20] Gerald AB. Failure of the mature hemodialysis arteriovenous fistula. Available online at: https://www.uptodate.cn/contents/zh-Hans/failure-of-the-mature-hemodialysis-arteriovenous-fistula? (Accessed February 25, 2026).

[21] Gerald AB. Risk factors for hemodialysis arteriovenous fistula failure. Available online at: https://www.uptodate.cn/contents/zh-Hans/risk-factors-for-hemodialysis-arteriovenous-fistula-failure? (Accessed February 25, 2026).

[22] Karen W. Arteriovenous fistula creation for hemodialysis and its complications. Available online at: https://www.uptodate.cn/contents/zh-Hans/arteriovenous-fistula-creation-for-hemodialysis-and-its-complications? (Accessed February 25, 2026).

[23] Salih SSM Mohamed KO Mohamedali AOO Mahmoud AAO Ibrahim DAS Abdallah KF. Predictors of early arteriovenous fistula failure in patients with end stage renal disease on hemodialysis: a systematic review and meta-analysis. Patient Saf Surg. (2025) 19(1):24. 10.1186/s13037-025-00449-9 40898218 PMC12403630

[24] Raza H Hashmi MN Dianne V Hamza M Hejaili F A-Sayyari A. Vascular access outcome with a dedicated vascular team based approach. Saudi J Kidney Dis Transpl. (2019) 30(1):39–44. 10.4103/1319-2442.252931 30804265

[25] Okano H Mayumi T Kataoka Y Banno M Tsujimoto Y Shiroshita A. Outcomes of simulation-based education for vascular access: a systematic review and meta-analysis. Cureus. (2021) 13(8):e17188. 10.7759/cureus.17188 34414052 PMC8365863

[26] Lopes JRA Marques ALB Correa JA. Randomised clinical study of the impact of routine preoperative Doppler ultrasound for the outcome of autologous arteriovenous fistulas for haemodialysis. J Vasc Access. (2021) 22(1):107–14. 10.1177/1129729820927273 32519569 PMC7897791

[27] Georgiadis GS Charalampidis DG Argyriou C Georgakarakos EI Lazarides MK. The necessity for routine pre-operative ultrasound mapping before arteriovenous fistula creation: a meta-analysis. Eur J Vasc Endovasc Surg. (2015) 49(5):600–5. 10.1016/j.ejvs.2015.01.012 25736517

[28] Niyyar VD Wasse H. Vessel mapping for dialysis access planning. Semin Dial. (2017) 30(4):305–8. 10.1111/sdi.12594 28382736

[29] Malovrh M. Vascular access creation and care should be provided by nephrologists. J Vasc Access. (2015) 16(Suppl 9):S20–3. 10.5301/jva.5000332 25751545

[30] Aitken E Kearns R Gaianu L Jackson A Steven M Kinsella J. Long-term functional patency and cost-effectiveness of arteriovenous fistula creation under regional anesthesia: a randomized controlled trial. J Am Soc Nephrol. (2020) 31(8):1871–82. 10.1681/ASN.2019111209 32709710 PMC7460891

[31] Ismail A Abushouk AI Bekhet AH Abunar O Hassan O Khamis AA. Regional versus local anesthesia for arteriovenous fistula creation in end-stage renal disease: a systematic review and meta-analysis. J Vasc Access. (2017) 18(3):177–84. 10.5301/jva.5000683 28478618

[32] Lok CE Allon M Moist L Oliver MJ Shah H Zimmerman D. Risk equation determining unsuccessful cannulation events and failure to maturation in arteriovenous fistulas (REDUCE FTM I). J Am Soc Nephrol. (2006) 17(11):3204–12. 10.1681/ASN.2006030190 16988062

[33] Nantakool S Rerkasem K Reanpang T Worraphan S Prasannarong M. A systematic review with meta-analysis of the effects of arm exercise training programs on arteriovenous fistula maturation among people with chronic kidney disease. Hemodial Int. (2020) 24(4):439–53. 10.1111/hdi.12875 32975044

[34] Wan Q Yang S Li L Chu F. Effects of far infrared therapy on arteriovenous fistulas in hemodialysis patients: a meta-analysis. Ren Fail. (2017) 39(1):613–22. 10.1080/0886022X.2017.1361835 28805538 PMC6446143

[35] Kanchanasuttirak P Pitaksantayothin W Saengprakai W Kanchanabat B. Systematic review and meta-analysis: efficacy and safety of balloon angioplasty in salvaging non-matured arteriovenous fistulas. J Vasc Access. (2023) 24(6):1244–52. 10.1177/11297298221085440 35389293

[36] Parisotto MT Schoder VU Miriunis C Grassmann AH Scatizzi LP Kaufmann P. Cannulation technique influences arteriovenous fistula and graft survival. Kidney Int. (2014) 86(4):790–7. 10.1038/ki.2014.96 24717298 PMC4184025

[37] Chow J Rayment G Miguel SS Gilbert M. A randomised controlled trial of buttonhole cannulation for the prevention of fistula access complications. J Ren Care. (2011) 37(2):85–93. 10.1111/j.1755-6686.2011.00211.x 21561544

[38] Li KJ Xiao YF Hu J Yu JY Tang LQ Shen XF. Meta-analysis of the application effect of ultrasound-guided technology in arteriovenous fistula cannulation in hemodialysis patients. Chin J Blood Purif. (2023) 22(12):949–54. 10.3969/j.issn.1671-4091.2023.12.014

[39] Turmel-Rodrigues L Pengloan J Rodrigue H Brillet G Lataste A Pierre D. Treatment of failed native arteriovenous fistulae for hemodialysis by interventional radiology. Kidney Int. (2000) 57(3):1124–40. 10.1046/j.1523-1755.2000.00940.x 10720965

